# Not Charity but Humanity

**Published:** 1919-12-13

**Authors:** 


### The Hospital

The Workers' Newspaper of Administrative Medicine and Institutional Life, Administration, National Insurance and Health.

No. 1749, Vol. LXVII. SATURDAY, DECEMBER 13, 1919. PRICE TWOPENCE

### NOT CHARITY BUT HUMANITY.

His Royal Highness the Prince of Wales, immediately after his return from Canada and the United States, has given evidence of the possession of an untiring energy which has won for him the envy of a multitude of the younger men amongst us. He has won a welcome so warm and universal as to make his advent a joy to everyone fortunate enough to share in the proceedings. Like his father he has really a great gift for speaking, and the Prince's speech at the Middlesex Hospital Dinner on Tuesday, the 9th inst., gave gratifying evidence of this fact. He declared he felt some diffidence in undertaking the task which lay before him, but the subject was so full of human interest that H.R.H. was encouraged to say what he felt about it in his own way. He declared—his words might helpfully find a place over the entrance to every hospital in the land—that "the claim of a "hospital is not charity; it is humanity—the "humanity which bids us help our fellows in distress." He declared that the welfare of our hospitals is a question which must appeal to all of us. He often wondered if the people understood what a hospital really is. Of course they regard it as a worthy and necessary institution; but that is merely looking at it above the surface. He wanted to break that surface that night and see the hospital from beneath, even though it be only a fleeting glance. If one were to ask the proverbial man in the street to define the word "hospital," the Prince wondered what the average answer would be. He thought it might be: "Why, it is a place where poor people go when they cannot afford a doctor." In a sense that was correct. At the same time, to be Irish, it is wrong. Take the case of the ordinary person in good health earning a comfortable living, and able to save something at the same time. Disaster, in the shape of disease or accident, befalls him. Were he to be treated at home, the chances are that his savings would be swallowed up. Instead of that, he is taken to the hospital, and it costs him nothing to be treated with the same skill that people of wealth have to pay hundreds of pounds for. This is one instance of hospital work, but it is not charity; it is humanity—the humanity which bids us help our fellows in distress.

Again, take the Out-patients' Department of any hospital. It is daily crowded with people in need of relief who are able to go to and fro. Again they benefit by the skill of untiring surgeons, doctors and nurses. Some go away, taking it as their due, and others give what they can as a mark of gratitude. To maintain properly a hospital such as the Middlesex Hospital a very large sum is required. At the moment £200,000 are urgently needed to place it on a proper basis. That is a vast sum, and it might seem almost impossible to acquire it. But what has been termed impossible has been successfully achieved and tackled before now. There is another purpose for which that sum is required. H.R.H. would remind us of a very important work that is done by the hospitals—that of training in successive generations medical men and sending them out on missions of health. "I would ask you also to remember that Middlesex Hospital has a special claim on account of the great fight that it is making against the dread disease of cancer."

We have given some material points from H.R.H.'s speech, because it was a specially good speech, and we wish to preserve it in The Hospital as a type for other Chairmen who come after. It is encouraging, as it is wonderful, that he should have so fully mastered the chief points which are material for the purpose of raising money, and exciting general sympathy for the Middlesex Hospital, and indeed for all voluntary hospitals. Our mission at this season is especially that of raising funds throughout Great Britain for our voluntary hospitals, who have ahead of them three lean years. We have already shown in these columns continuously that all the voluntary hospitals throughout the country need, and are entitled to have given to them, at this time much larger revenues than they at present possess. In another column we have endeavoured to show a way whereby the whole money needed may be speedily forthcoming. As the Prince properly urged, if the hospital is to carry on, it needs people to realise that the hospital is a very necessary thing for the welfare of the nation and every individual in it. That is one reason why it needs money. The London hospitals are more fortunate than their brothers throughout the country, as they receive substantial and increasing financial support year by year from gifts from the three Hospital Funds, as well as from legacies and other gifts from the general public. What the nation requires for the sake of its healthy residents, as well as for the sick, is a lively appreciation of the extent to which the Governors of our hospitals are harassed by lack of funds. Would that it were possible to make every intelligent member of the community understand and realise the terrible strain that is brought about by lack of money, a want which seriously handicaps the advancement of medical science.

Add to this knowledge the remembrance of the twofold fact that every principal hospital, and most of the small ones, during the war have had passed through their hands many thousands of wounded men, many of whom on demobilisation and discharge from the hospitals, have gone home to their own districts, which contain hospitals, in whose aid they can declare from experience, their great and indispensable value to the community. Remember Liverpool's example to provide with all possible speed an adequate sum of money to tide each hospital over the three lean years ahead. Finally remember the Prince's speech. May it, as we have no doubt it will, prove of supreme value not only to the Middlesex but to every hospital throughout Great Britain.

